# HLA-E expression constitutes a novel determinant for ALL disease monitoring following hematopoietic stem cell transplantation

**DOI:** 10.1038/s41409-021-01231-y

**Published:** 2021-03-03

**Authors:** Sarah B. Reusing, Angela R. Manser, Stefanie Groeneveld-Krentz, Vera Rebmann, Peter A. Horn, Roland Meisel, Leonid Karawajew, Arndt Borkhardt, Markus Uhrberg, Florian Babor

**Affiliations:** 1 Institute for Transplantation Diagnostics and Cell Therapeutics, Heinrich Heine University, Düsseldorf, Germany; 2 Department of Pediatric Oncology, Hematology and Clinical Immunology, Center for Child and Adolescent Health, Medical Faculty, Heinrich-Heine University, Düsseldorf, Germany; 3 Department of Paediatric Oncology/Hematology, Charité Universitätsmedizin Berlin, Berlin, Germany; 4 Institute for Transfusion Medicine, University Hospital Essen, Essen, Germany; 5 Division of Pediatric Stem Cell Therapy, Clinic for Pediatric Oncology, Hematology and Clinical Immunology, Center for Child and Adolescent Health, Medical Faculty, Heinrich-Heine University, Düsseldorf, Germany

**Keywords:** Bone marrow transplantation, Acute lymphocytic leukaemia

**To the Editor:**

Clinical and experimental evidence suggests that acute lymphoblastic leukemia (ALL) is highly resistant to natural killer (NK) cell-mediated killing, even in the allogeneic setting [1, 2]. The mechanisms mediating this resistance are poorly understood but are thought to involve engagement of inhibitory killer immunoglobulin-like receptors (KIR) by HLA-B and HLA-C-encoded ligands, as well as the inhibitory interaction of the NKG2A receptor with HLA-E on leukemic cells [3–5]. Paradoxically, we have recently shown that HLA-E surface expression is specifically downregulated on ALL blasts at the time of diagnosis, whereas residual non-leukemic B cells maintained normal HLA-E expression levels [6].

In order to further explore the diagnostic relevance of HLA-E as a novel marker for disease monitoring, we followed the expression of HLA-E through serial time-points during the disease history of pediatric ALL patients. We started by retrospectively analyzing a cohort of 23 patients diagnosed with precursor B-ALL and for whom samples had been collected at the time of diagnosis, during and after chemotherapy (Supplementary Table S1). In accordance with our previous study, HLA-E downregulation was detected in all patients on leukemic blasts at the time of diagnosis (Fig. 1a, b, and f) but not on residual B cells (data not shown). In patients achieving complete remission following the induction phase (*n* = 17) HLA-E levels were restored to normal ranges and stayed within this range during the observation time (Fig. 1a, f), reflecting the absence of leukemic blasts. In all cases of relapse (*n* = 6), restored HLA-E levels after the induction phase dropped significantly indicating relapse (Fig. 1b, f). As expected, individuals homozygous for genotype *01:03 showed the strongest HLA-E surface expression. This held true in healthy individuals and in currently and recently [6] investigated ALL patients (Suppl. Fig. S1). Interestingly, downregulation of surface HLA-E was not accompanied by an increase of plasma soluble (s)HLA-E levels, which remained comparable to healthy individuals (Suppl. Fig. S2). In contrast, there was a significant correlation between the percentage of blasts and the downregulation of HLA-E (*p* = 0.046, Suppl. Fig. S3).

**Fig. 1:**
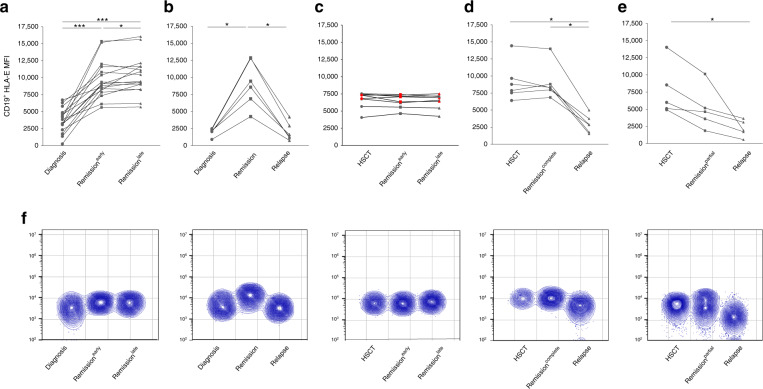
Consistent downregulation of HLA-E among patients with relapse following chemotherapy or transplantation. Leukemic B cells were stained with HLA-E specific human monoclonal antibody (3D12) and identified via CD19 expression and side scatter. Measurements of all time points were made with identical instrument settings. **a** HLA-E expression on B cells (CD19^+^ subset) in individual patients treated with chemotherapy without or (**b**) with relapse at indicated time points (time from diagnosis to early remission 443–826 days, time from diagnosis to late remission 980–1691 days), (**c**) HLA-E expression on B cells (CD19^+^ subset) in patients in remission after transplantation. **d** HLA-E expression on B cells (CD19^+^ subset) in patients following transplantation with relapse following complete remission or (**e**) relapse following incomplete remission. **f** Flow cytometric data (concatenate contour plots) are shown for representative leukemic patients included in panels **a**–**e**, respectively. Statistical significance was determined by one-way-Anova or paired t-test (*P < 0.05, ***P < 0.001).

We next evaluated HLA-E surface expression in 19 patients undergoing hematopoietic stem cell transplantation (HSCT). Initial values were within the range of the respective stem cell donors (data not shown). Again, patients who achieved long-term remission following HSCT (*n* = 8) stably maintained HLA-E expression levels comparable to initial values (Fig. 1c, f). In contrast, HLA-E expression dropped significantly in relapsed patients, again reflecting recurrence of leukemic blasts (*n* = 11). Among these, six patients with late relapse (>6 months after HSCT) managed to reach reconstituted HLA-E levels initially after HSCT but eventually experienced HLA-E downregulation at the time of relapse (Fig. 1d, f). Finally, five patients who relapsed early (<6 months after HSCT) showed rapid HLA-E downregulation during the partial remission phase followed by a further drop accompanying relapse (Fig. 1e, f).

In order to address the functional relevance and diagnostic potential of the observed HLA-E downregulation, we next studied the cases of three ALL patients undergoing two consecutive HSCTs in more detail (Fig. 2a–d). HLA-E levels remained stable for 178–372 days post 1st HSCT. During this period, minimal residual disease (MRD) levels in BM and PB, measured by PCR (<1 × 10^−3^) and by flow-cytometry [7] (0.00%) suggested a stable molecular remission. Notably, a subsequent decrease of HLA-E expression occurred in parallel to an increasing MRD load of ≥10^−3^ (PCR) and >0.01% (flow-cytometry), marking the onset of molecular relapse. Once having achieved a further remission, similar impacts on cell surface expression could be observed: following 2nd HSCT, HLA-E levels dropped significantly accompanied by increasing MRD loads. These observations appeared to be independent of the conditioning regimen, donor type, graft-versus-host disease (GvHD) prophylaxis, and presence of GvHD. Furthermore, the correlation was also independent of the HCMV status of the patients (Table S1).

**Fig. 2:**
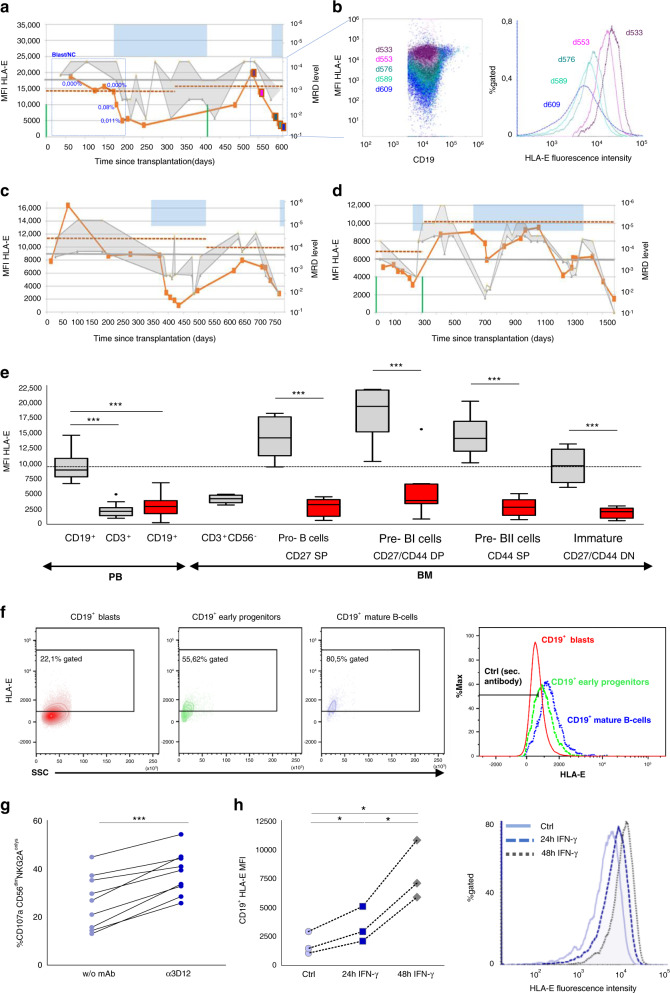
Leukemic blasts downregulate HLA-E expression to low but tolerogenic levels, which inhibits NK cell activation and correlates with a reoccurrence of the disease. **a**, **c**, **d** Comparison of HLA-E levels and MRD values in three representative patients undergoing multiple allogeneic HSCTs (gray shaded area: range between minimum and maximum detected PCR MRD levels). The three patients were observed for 1.7–4.3 years by analyzing consecutive PBMC samples and results were correlated with analyses of minimal residual disease (MRD) monitoring (PCR and flow-cytometry) in bone marrow samples. Blue shaded area: indicating antineoplastic treatment modalities (chemotherapy, antibody therapy, CIK cell infusion); vertical green: time point of HSCT, red line: HLA-E surface expression; horizontal gray line: PCR MRD threshold; blue dots: flow-cytometry MRD levels; horizontal dashed red line: HLA-E level of the respective donor. **b** Dot plot (left panel) and histogram (right panel) overlays showing downregulation of HLA-E expression on CD19^+^ cells from one representative ALL patient at different time points from transplantation to relapse (day +533 violet; day +553 pink; day +576 dark green; day +589 light green; and day +609 blue). **e** HLA-E surface expression levels of B cell precursor stages in bone marrow aspirates. Box plots showing staining of HLA-E surface expression on peripheral blood (PB) CD19^+^ and CD3^+^ lymphocytes from 29 healthy volunteers (HV: gray), 40 diagnosed ALL patients (ALL: red, initial and relapse samples) and on bone marrow cells from 8 healthy volunteers (gray) and 8 ALL patients (red). b cell precursor stages in bone marrow aspirates were defined as previously reported in Vaskova et al. [8]. The dotted black baseline indicates the mean expression level of HLA-E on CD19^+^ peripheral blood cells from healthy volunteers. Error bars represent the standard error of the mean. Statistical significance was determined by a Mann–Whitney U test or unpaired t-test (***p < 0.001). **f** HLA-E surface expression levels of CD19^+^ blasts, early progenitors (hematogones) and more mature CD19^+^ B cells. Cell populations and maturity stages were distinguished according to flow MRD standards published by Karawajew et al. [7]. Histogram overlay (right panel) showing downregulation of HLA-E expression on leukemic blasts compared to normal CD19^+^ cells. **g** To further elucidate whether suppression of inhibitory signals could enhance NK cell-mediated anti-tumor responses, NKG2A/HLA-E interaction was blocked via αHLA-E (3D12, gray bar). Blocking antibody and cytolytic activity was measured via CD107a mobilization assay. CD107 frequency was measured exclusively on KIR^−^NKG2A^+^CD56^dim^ NK^−^-cells. (h) To determine whether HLA-E downregulation was a reversible process, ALL samples (*n* = 3) were cultured in the presence or absence of IFNγ (100 U/mL) and HLA-E expression was measured at the indicated time points (left panel). The respective histograms of a representative experiment (right panel) at 0 h (solid violet), 24 h (dashed blue), and 48 h (dotted gray). Statistical significance was determined by one-way-Anova or paired t-test (*p < 0.05, **p < 0.01, ***p < 0.001).

In the next step, we wanted to address the question of whether HLA-E downregulation on peripheral leukemic blasts reflects the degree of expression on immature precursor B cells found in BM samples or is a leukemia-specific mechanism, possibly in order to escape immune surveillance. For this purpose, we assigned each ALL to a specific stage of B cell development according to the expression of stage-specific markers [8] and directly compared their HLA-E levels to those of the respective healthy precursor B cell stages found in BM samples (Fig. 2e). First of all, peripheral ALL blasts exhibited a similar degree of HLA-E downregulation compared to ALL blasts found in BM. More importantly, leukemic HLA-E levels were significantly lower than those of the respective healthy precursor B cell stages in BM (Fig. 2e). In the next step, we distinguished in BM between mature and immature B cells (early precursor hematogones), and leukemic blasts within patient samples by staining for flow-cytometry MRD [7] (Fig. 2f). We found that the HLA-E surface expression on early precursor B cells was significantly lower than on more mature cells. In comparison, the lowest values for HLA-E could be found on leukemic blasts. The data thus suggest leukemia-specific downregulation of HLA-E expression on blasts independent of the underlying B cell precursor maturity stage.

To gain mechanistic insights into the paradoxical finding of HLA-E downregulation, which makes leukemic blasts principally more susceptible to NK cell-mediated killing, functional experiments were performed: blockade of the NKG2A/HLA-E interaction by using an anti-HLA-E (3D12) mAb increased the cytolytic activity of healthy NK cells significantly, making leukemic blasts susceptible to recognition by NKG2A^+^ NK cells (Fig. 2g). The data demonstrate that the low level of HLA-E, present on ALL blasts, is still sufficient to induce an inhibitory signal through CD94/NKG2A and consequently HLA-E appears to still protect leukemic cells from NK cell-mediated lysis. Importantly, HLA-E downregulation on leukemic blasts could be reversed by IFNγ treatment, with full restoration of HLA-E surface expression after 48 h (Fig. 2g), documenting that the HLA-E gene is not permanently silenced.

Presently, qPCR from bone marrow biopsies represents the most validated and standardized method for MRD detection, which significantly correlates with clinical outcomes in leukemia [9]. The present analyses suggest that flow cytometric quantification of HLA-E expression on leukemic blasts provides a novel promising tool for ALL monitoring in peripheral blood complementing previously established ways to monitor the leukemic burden such as BM-based molecular MRD monitoring. Importantly, no HLA-E deletion variants were detected in any of the ALL samples analyzed so far. Moreover, HLA-E is genetically highly conserved with only two very similar allelic variants (Table S1) [10] that are picked up by the same specific mAb. Based on the data from this and a previous study, together comprising >50 different ALL cases, HLA-E constitutes a highly valuable novel flow MRD marker, that is common to all ALL cases analyzed so far and is thus likely to be applicable to virtually all ALL cases. It has to be noted, that the present data have to be validated in a prospective study hopefully leading to an algorithm quantitatively defining critical HLA-E levels that are predictive for MRD. This could provide the basis for a novel strategy in which regular flow cytometric analysis of HLA-E from small blood samples (e.g., <500 µl from finger-prick blood) could potentially improve ALL monitoring by providing a reliable ‘alert’ signal for subsequent validation by molecular, bone marrow-based MRD assessment.

Mechanistically, the study suggests strong selective pressure for expression of low but still tolerogenic HLA-E levels on ALL blasts, in turn indicating tight surveillance of ALL by NKG2A^+^ NK cells. The functional data, demonstrating restored killing of ALL blasts by specific blocking of HLA-E/NKG2A interaction, suggest that HLA-E-mediated resistance to host immune surveillance is only given within a small corridor enabling leukemic escape from both T cell and NK-mediated control. Moreover, they challenge the prevailing view that B-ALL is generally resistant to NK cell surveillance. In fact, cellular therapy combining allogeneic NK cells with antibody-mediated blocking of the HLA-E/NKG2A inhibitory axis, as provided by the NKG2A-specific therapeutic reagent Monalizumab, could open novel avenues for treatment of B-ALL [11, 12].

## Supplementary information

SUPPLEMENTAL MATERIAL
