# Supplementary material for: HLA-E expression constitutes a novel determinant for ALL disease monitoring following hematopoietic stem cell transplantation
Source: Bone Marrow Transplant. 2021 Mar 3;56(7):1723–7. doi: 10.1038/s41409-021-01231-y (PMC8263342; doi:10.1038/s41409-021-01231-y)
Supplement: Supplementary file 1 — SUPPLEMENTAL MATERIAL [file 41409_2021_1231_MOESM1_ESM.docx]

Supplement

**Material and methods**

**Patients and controls**

Peripheral blood (PB) was obtained from 34 pediatric patients with a) newly diagnosed B-precursor ALL, b) relapse and (c) after hematopoietic stem cell transplantation (HSCT; age, mean 7.4 years) who were treated at the University Hospital in Duesseldorf. Follow-up samples were taken from all patients undergoing chemotherapy or HSCT. PB samples from the respective stem cell donor served as controls.

**HLA-E staining**

For HLA-E surface staining frozen PBMCs were incubated with unlabeled primary antibody to HLA-E (3D12), followed by FITC-conjugated anti-mouse IgG (BioLegend). Free secondary antibody-binding sites were blocked with staining buffer (PBS, 0,5%w/v BSA, 2mM EDTA) before further staining of primary cell samples with directly conjugated mAb.

**IFN-γ stimulation**

To examine the effect of interferon (IFN)-γ on HLA-E antigen expression, cells were treated in culture with IFN-γ (100 U/mL) for 24 and 48 h, after which cell-surface HLA-E antigen expression was examined as described above.

**HLA-E blocking assay and NK cell degranulation**

Thawed PBMC from healthy donors were cultured overnight in RPMI 1640 containing 10% fetal bovine serum, 5% human serum type AB (Biochrom), and 500 U/mL interleukin-2 (Novartis, Basel, Switzerland). For blocking experiments leukemic blasts were incubated with or without HLA-E antibody at a concentration of 5µg/mL, 20 min prior to incubation. PBMC and ALL blasts were then mixed at an effector/target (E/T) ratio of 10:1 in a volume of 200 μL in a 96-well plate. For analysis of cytotoxic granule mobilization, CD107a monoclonal antibody was added prior to incubation. After incubation for 1 h, 2 μL of 2 mM Monensin (Biolegend) were added and incubated for a further 5 h. Finally, the cells were washed in phosphate-buffered saline (Lonza) and stained with monoclonal antibodies (CD56, CD3, NKG2A and KIR-Mix (CD158a/h/g, CD158b1/b2/j, CD158e1).

| UID | immunophenotype | disease state | sex | age (years) | CMV status | CMV donor | HLA-E genotype | relapse |
| --- | --- | --- | --- | --- | --- | --- | --- | --- |
| 1 | pre-B ALL | ini | m | 12,0 | neg |  | GR | no |
| 2 | pre-B ALL | ini | m | 6,9 | neg |  | GR | no |
| 3 | pre-B ALL | ini | f | 11,6 | unknown |  | RR | no |
| 4 | early pre B-ALL | ini | m | 7,4 | pos |  | GR | no |
| 5 | common ALL | ini | m | 16,1 | neg |  | GG | no |
| 6 | common ALL | ini | m | 5,8 | pos |  | GR | no |
| 7 | common ALL | ini | f | 13,5 | neg |  | RR | no |
| 8 | pre-B ALL | ini | f | 1,3 | neg |  | RR | no |
| 9 | common ALL | ini | m | 0,9 | neg |  | RR | no |
| 10 | common ALL | ini | f | 3,3 | neg |  | GR | no |
| 11 | common ALL | ini | m | 8,5 | neg |  | GR | no |
| 12 | common ALL | ini | f | 3,1 | neg |  | RR | no |
| 13 | common ALL | ini | m | 2,1 | neg |  | unknown | no |
| 14 | common ALL | ini | m | 3,7 | neg |  | GG | no |
| 15 | pre-B ALL | ini | m | 3,5 | pos |  | RR | no |
| 16 | common ALL | ini | f | 7,4 | neg |  | GG | no |
| 17 | common ALL | in | f | 16,5 | neg |  | RR | no |
| 18 | common ALL | ini | f | 1,6 | neg |  | GR | yes |
| 19 | common ALL | ini | m | 2,2 | neg |  | GR | yes |
| 20 | pre-B ALL | ini | m | 1,2 | pos |  | GR | yes |
| 21 | common ALL | HSCT | m | 2,6 | neg | pos | RR | yes |
| 22 | pre-B ALL | ini/HSCT | f | 17,6 | pos | pos | GR | yes |
| 23 | pre-B ALL | ini/HSCT | m | 4,4 | pos | neg | GR | yes |
| 24 | common ALL | HSCT | m | 12,7 | pos | pos | RR | no |
| 25 | pre-B ALL | HSCT | m | 3,0 | neg | pos | GG | no |
| 26 | common ALL | HSCT | f | 13,2 | neg | neg | GG | no |
| 27 | early pre B-ALL | ini/HSCT | f | 6,9 | pos | pos | GG | no |
| 28 | common ALL | HSCT | m | 5,1 | pos | pos | GR | yes |
| 29 | common ALL | HSCT | f | 18,0 | neg | pos | GR | yes |
| 30 | pre-B ALL | HSCT | f | 2,5 | neg | pos | RR | yes |
| 31 | early pre B-ALL | HSCT | m | 17,3 | neg | neg | RR | yes |
| 32 | common ALL | HSCT | m | 4,0 | neg/pos | pos/pos | GR | yes |
| 33 | common ALL | HSCT | m | 5,9 | pos/neg | neg/pos | GR | yes |
| 34 | common ALL | HSCT | f | 7,8 | neg/pos | pos/neg | GR | yes |

**Table S1. Patient and disease characteristics, genetics and outcome of children with B-Cell Precursor ALL**. HLA-E genotype was defined according to Geraghty et al., 1992.

ALL: acute lymphoblastic leukemia, ini: initial diagnosis, HSCT: hematopoietic stem cell transplantation

**Figure legends**

**Figure S1:** Surface expression in 64 healthy controls (HV) and 23 ALL patients. The fold expression level in different HLA-E genotype groups was compared by unpaired t test, *p<0.1, **p<0.01, ***p<0.001.

**Figure S2:** (a) Levels of soluble sHLA-E have been analyzed in plasma samples from 20 healthy volunteers (HV), 60 ALL patients at the time of diagnosis/relapse and/or in remission. Horizontal lines indicated medians. Results are expressed as pg/mL. (b) Correlation between soluble sHLA-E and surface expression levels of all ALL patients at the time of diagnosis/relapse (circle, blue) and/or remission (rhombus, light blue).

**Figure S3:** Correlation between MFI and % CD19+CD45-leukemic cells (linear regression model; p=0.046)


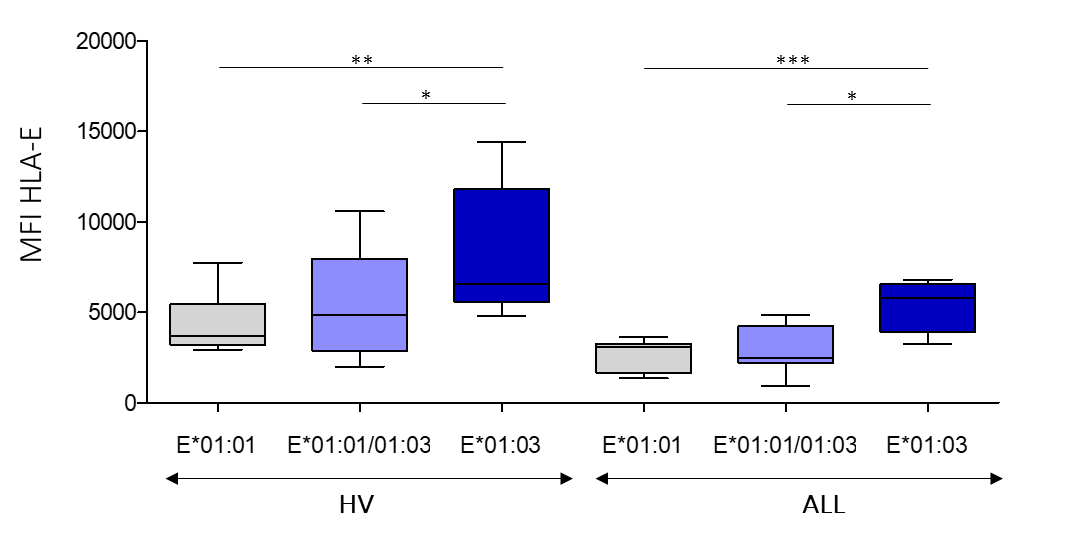


Figure S1


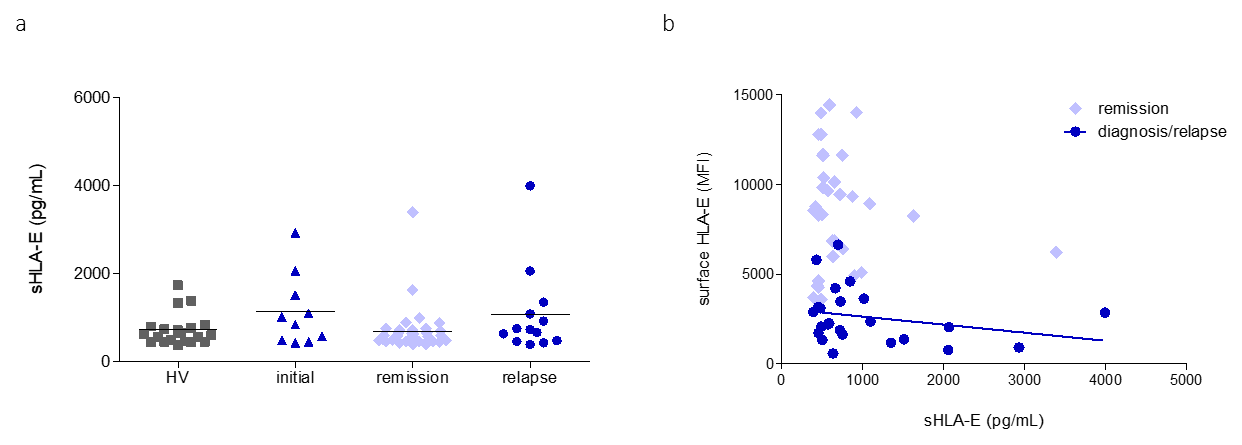


Figure S2


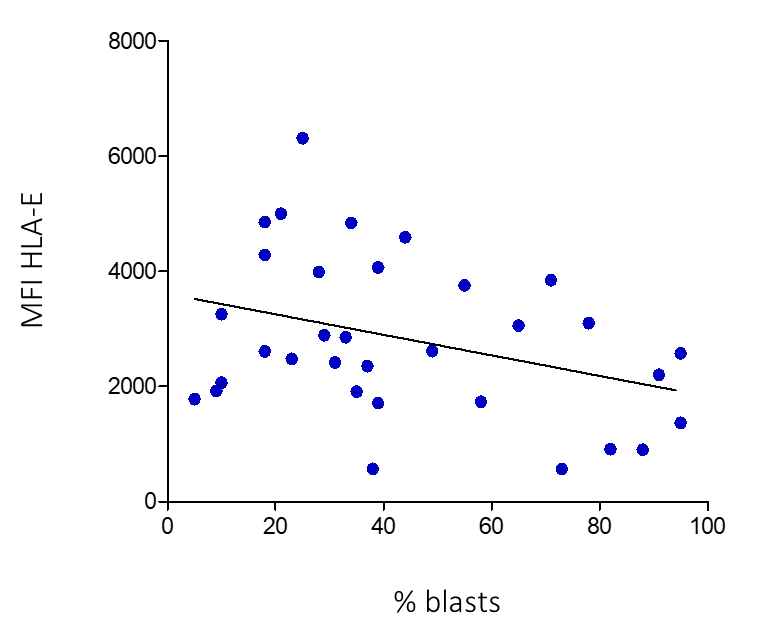


Figure S3
